# Overview of Deep Learning and Nondestructive Detection Technology for Quality Assessment of Tomatoes

**DOI:** 10.3390/foods14020286

**Published:** 2025-01-16

**Authors:** Yuping Huang, Ziang Li, Zhouchen Bian, Haojun Jin, Guoqing Zheng, Dong Hu, Ye Sun, Chenlong Fan, Weijun Xie, Huimin Fang

**Affiliations:** 1 College of Mechanical and Electronic Engineering, Nanjing Forestry University, Nanjing 210037, China; liziang@njfu.edu.cn (Z.L.); bzc@njfu.edu.cn (Z.B.); 15398254488@163.com (G.Z.); fancl@njfu.edu.cn (C.F.); weijun_xie@njfu.edu.cn (W.X.); 2 School of Flexible Electronics (Future Technologies) and Institute of Advanced Materials (IAM), Nanjing Tech University, Nanjing 211816, China; iamhjjin@njtech.edu.cn; 3 College of Optical, Mechanical and Electrical Engineering, Zhejiang A&F University, Hangzhou 311300, China; hudong538338@zju.edu.cn; 4 College of Food Science and Light Industry, Nanjing Tech University, Nanjing 211816, China; sunye@njau.edu.cn; 5 School of Agricultural Engineering, Jiangsu University, Zhenjiang 212013, China; fanghuimin@ujs.edu.cn

**Keywords:** tomato quality, nondestructive detection techniques, deep learning, applications

## Abstract

Tomato, as the vegetable queen, is cultivated worldwide due to its rich nutrient content and unique flavor. Nondestructive technology provides efficient and noninvasive solutions for the quality assessment of tomatoes. However, processing the substantial datasets to achieve a robust model and enhance detection performance for nondestructive technology is a great challenge until deep learning is developed. The aim of this paper is to provide a systematical overview of the principles and application for three categories of nondestructive detection techniques based on mechanical characterization, electromagnetic characterization, as well as electrochemical sensors. Tomato quality assessment is analyzed, and the characteristics of different nondestructive techniques are compared. Various data analysis methods based on deep learning are explored and the applications in tomato assessment using nondestructive techniques with deep learning are also summarized. Limitations and future expectations for the quality assessment of the tomato industry by nondestructive techniques along with deep learning are discussed. The ongoing advancements in optical equipment and deep learning methods lead to a promising outlook for the application in the tomato industry and agricultural engineering.

## 1. Introduction

### 1.1. Tomatoes

Tomatoes (*Solanum lycopersicum*) are globally recognized as the most valuable fruit and horticultural crop species [1] due to their unique flavor and abundant nutrients [2]; particularly, the lycopene has the potential to reduce cardiovascular diseases [3] and ameliorate various chronic degenerative diseases [4]. In 2022, the global production of tomatoes reached 186.11 million tons and China was the largest tomato producer with 68.34 million tons in the world. Additionally, in 2003, the tomato harvested area in China was 0.875 million hectares, while it reached 1.142 million hectares in the year of 2022, representing an increase of 2.67 million hectares compared with 2003 [5]. These indicate a substantial expansion in the scale of tomato planting, suggesting that the tomato industry in China is undergoing significant growth and development. As living standards increase, consumers are willing to pay more for delicious and high-quality vegetables and fruits [6]. Tomatoes are susceptible to spoilage during the post-harvest period due to collision and extrusion; thus, the quality assessment plays a key role in detection and grading after harvest [7]. Due to the subjective form of assessment and the use of destructive detection, the assessment of tomato quality in the past has been difficult and time-consuming. Nondestructive detection technology, with the advantages of fast speed, low cost, and the ability to perform real-time online detection, is capable of avoiding the loss of composition and nutrition in samples. Therefore, nondestructive detection technology is of paramount significance for the optimization of the tomato industry and the enhancement of market competitiveness [8].

### 1.2. Nondestructive Detection Technology and Deep Learning

The physical, chemical, and biological properties of agricultural products could be measured without causing damage to the integrality of samples by employing nondestructive detection techniques based on characteristics of light, sound, electricity, magnetism, and force. According to their respective characteristics, nondestructive detection techniques could be commonly classified into three principal categories, i.e., mechanical characterization, electromagnetic characterization, and electrochemical sensors. Briefly, mechanical-based techniques assess quality attributes based on the mechanical or acoustic response of the fruit to some excitation such as knocks and sound waves [9]. Electromagnetic-based techniques evaluate quality parameters based on the interaction of fruit tissues with electromagnetic waves [10], while electrochemical sensor techniques measure quality indicators in accordance with the chemical reaction of specific components of the fruit by the sensor [11,12,13,14].

However, a significant challenge in current nondestructive techniques is the processing of vast amounts of data. The simple data structure and relatively uncomplicated mathematical models of machine learning algorithms often limit the application of agricultural products for the extraction of diverse features [15,16]. The growth in computing power and the accessibility of vast datasets led to the emergence of a prominent and promising area of machine learning, deep learning, which employs artificial neural networks to address intricate challenges [17]. Numerous studies in the literature have demonstrated that the integration of deep learning into agricultural engineering could provide a great potential to assess the quality attributes of agricultural products more intelligently, conveniently, and in a standardized way using nondestructive detection techniques [18,19]. Tomato, as an essential fruit or vegetable for daily life, is normally evaluated by nondestructive techniques coupled with machine learning [20,21]. However, previous studies focused on the nutritional value of tomatoes [4,22,23], disease detection in tomato fruits and plants [24,25], tomato cultivation techniques [26,27,28], tomato defect and quality detection [29,30], and gene expression in tomatoes [31,32,33]. Few studies provide a comprehensive summary of tomato quality assessment using nondestructive detection techniques, especially for the combination of the ones with deep learning.

Therefore, the aim of this paper is to summarize the quality assessment of tomatoes based on nondestructive detection techniques with deep learning from the early 2000s. Frequently used nondestructive techniques are summarized into three categories based on mechanical, electromagnetic, and electrochemical properties. The mechanical technology encompasses a range of methodologies, such as mechanical loading, impact, acoustic waves, and ultrasound, whereas machine vision, spectroscopy, optical property, X-ray, and nuclear magnetic resonance techniques are prominent and widely used techniques in the domain of electromagnetic technology. Electronic noses and electronic tongues are typical electrochemical technologies that utilize electrochemical sensors to achieve quality evaluation. Additionally, their fundamental principles and applications in tomato quality assessment are introduced and discussed; moreover, the advantages and drawbacks of nondestructive techniques are compared. Advanced deep learning algorithms, coupled with nondestructive techniques with a high expectation of good detection performance for tomato analysis, are also summarized. Finally, the challenges and prospects of nondestructive techniques with deep learning are analyzed.

## 2. Bibliographic Search

The literature review is accomplished using two scientific databases, Web of Science and Google Scholar. The keywords used (in different combinations) include ‘tomato’, ‘cherry’, ‘cherry tomato’, ‘solanum lycopersicum’, ‘lycopersicum’, ‘detection’, ‘inspection’, ‘testing’, ‘assessment’, ‘evaluation’, ‘nondestructive’, ‘noninvasive’, ‘noncontact’, ‘deep learning’, ‘machine learning’, ‘neural network’, and ‘network’. Once the literature is retrieved, the following criteria are used to select the literature. (1) Tomatoes should be the subject for investigation rather than tomato products such as tomato sauce. (2) The paper is focused on post-harvested tomatoes instead of tomato plants or tomato growth processes. (3) At least one of the tomato qualities is mentioned, in other words, the study excludes research about simply identifying whether the object is a tomato or not. (4) The research papers related to tomato quality analysis in the past two decades were comprehensively retrieved.

## 3. Quality Assessment

Tomato, as one of the most popular vegetables in the world, has many nutrients including dietary fiber, organic acids, vitamin C, lycopene, potassium, and others. Quality assessment is necessary in order to enhance customer satisfaction and marketing competitiveness. Tomato quality generally involves external and internal qualities, which refer to the combination of the properties, attributes, or basic parameters of an object that have significance in determining the acceptance of consumers [34]. The external quality parameters like color, shape, size, maturity, and external defects are immediately apparent. The internal quality parameters, such as texture, soluble solids content (SSC), pH, moisture content, nutrients, internal defects, and so on, directly influence the post-harvest shelf life, flavor, and taste. If the tomato product is determined to be of good quality, that means it meets consumer expectations in terms of qualities.

The surface color and size of tomatoes are the key points of the outer appearance. According to the standards of the Ministry of Agriculture of China, tomatoes are divided into early (10%~30%), middle (40%~60%), and late (70%~100%) maturity stages based on the red surface area, and also classified into large (L > 7 cm), medium (5 cm < M < 7 cm) and small (S < 5 cm) specifications according to the transverse diameter. Moreover, the United States Department of Agriculture classifies tomatoes as green, breakers, turning, pink, light red, and red according to ripeness. During the tomato ripening process, the color of the skin changes prominently, which is primarily attributed to the accumulation of lycopene. Consequently, some scholars employed the CIELab system to determine the L*, a*, and b* values of tomato, thereby revealing that the a*/b* value is not only closely associated with the lycopene content but also could be utilized to classify the ripening stage of fresh tomatoes [35,36,37,38]. External quality can be directly observed through vision; thus, machine vision combined with advanced deep learning algorithms is the mainstream nondestructive means [39]. Internal quality parameters are crucial at harvest time, which not only determine the flavor and contribute to the sensory experience but also indicate the storage period and nutritional value of tomatoes [40]. A tomato with a higher firmness level correlates with a longer shelf life, which is essential for storage and transportation. Sugar and acid content are important factors that directly affect the flavor of tomato fruit. Total soluble solids (TSSs), the ratio of TSSs to titratable acid (TA), and the total sweetness index (TSI) are three indicators used to evaluate tomato flavor. TSSs reflect the dry matter content with the range of TSSs from 3 to 7% for tomato and 9 to 15% for cherry tomato fruit. Moreover, the ratio of TSSs to TA with the value of 12.5 for tomato is assessed as delicious. The TSI is used to indicate sweetness, which is calculated by assigning specific weight factors to sucrose (1.0), glucose (0.76), and fructose (1.5). Thus, a tomato with good quality should have both great external and internal qualities.

Current quality detection methods in the tomato industry primarily depend on manual grading and destructive sampling techniques, which are labor-intensive, time-consuming, and prone to subjective bias [41]. The external quality parameters of tomatoes, including size and weight, are typically measured using instruments such as calipers and scales. Color and external defects are usually observed visually, which are dependent on the experience of the workers and therefore inevitably lead to errors. The internal quality parameters of tomatoes, including firmness, SSC, acidity, pectin, and lycopene are normally analyzed by traditional detection methods through the classical Magness–Taylor firmness method [42], the refractometer [43], the pH meter [44,45], the part-by-part extraction method [46], and the liquid chromatography method [47]. These traditional methods primarily rely on physical or chemical analysis. However, such methods inevitably compromise the integrity of the sample, rendering them lossy detection methods. These limitations underscore the importance of nondestructive detection techniques that can simultaneously assess external and internal quality parameters with high accuracy and efficiency. Thus, the capability of nondestructive techniques for comprehensive quality assessment not only enhances the value and marketability of tomatoes but also aligns with consumer preferences for high-quality produce. By adopting such techniques, the tomato industry has the potential to achieve more objective, reliable, and efficient quality control to meet the diverse demands of modern markets.

## 4. Principle and Application

A range of nondestructive techniques are employed to assess the quality and properties of tomatoes without causing any physical damage. The nondestructive detection technique ensures the integrity of the fruit so as to further storage, transportation, and sales while minimizing waste. Table 1 summarizes the application for assessing tomato quality by various nondestructive techniques and reveals that electromagnetic technologies, particularly for Vis/NIR spectroscopy, showed exceptional precision and high correlation coefficients for assessing various quality parameters of tomatoes, such as SSC, lycopene, and β-carotene. Multispectral and hyperspectral imaging techniques showed varying detection performance depending on the mathematical models and quality parameters. Mechanical techniques and electrochemical sensors are skilled in measuring physical properties and freshness, demonstrating that the combination of different techniques and methods can provide comprehensive and accurate quality assessments.

### 4.1. Mechanical Characteristic Technology

**Figure 1:**
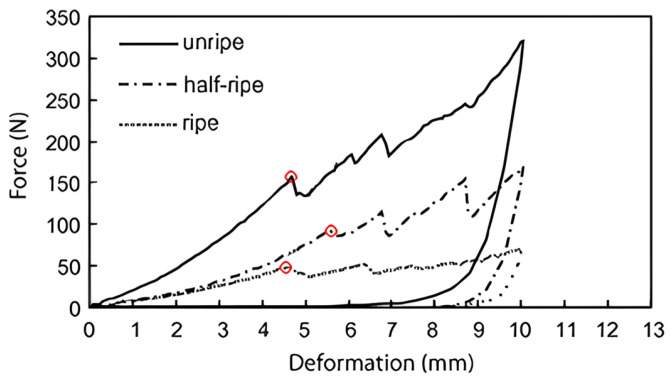
Typical force–deformation curve of tomatoes at three maturity levels with peak impact force (marked in red circles) using a free fall device to achieve impact test.

The mechanical nondestructive detection technology enables mechanical loading, impact, and sound waves to investigate elasticity, firmness, and other quality parameters of the object [81]. The mechanical loading and impact values are commonly analyzed by Hertzian theory, which demonstrates that the compressive stress between two bodies in contact is directly proportional to their modulus of elasticity and inversely proportional to their radius. In quasi-static compression tests, a metal probe or a metal disc is applied to induce micro-deformations of the sample through a small force to obtain the force/deformation curve [82]. Impact tests commonly use a probe to impact the sample or make the sample directly free fall, and the firmness value is obtained by the impact response wave. Quasi-static compression and impact tests combined with Hertzian theory are normally used to assess the firmness of the object. Lien et al. [49] employed a free fall device to assess the ripeness of tomatoes. Tomatoes fell freely on the piezoelectric sensor, which generated an analog signal proportional to the impact force of falling tomatoes. As shown in Figure 1, the peak impact forces were 161.2 N for unripe tomatoes, 89.4 N for half-ripe tomatoes, and 50.1 N for ripe tomatoes, respectively, which demonstrated that the force–deformation curve was capable of distinguishing the ripeness of tomatoes. Although the force–deformation measurement method exhibits low detection costs, the firmness obtained from this method only reflects the local firmness of the objects. [83].

In order to meet the desire of measuring overall quality in samples, an acoustic-based nondestructive detection technique was applied to exploit reflection characteristics, the attenuation coefficient, propagation speed, and natural frequency, to assess quality attributes. Similar to the impact test, acoustic measurement employs a plastic probe to gently tap the sample, and the acoustic signals traveling across the sample are acquired, which are related to overall features of the sample. The relationship between acoustic signals and elastic properties of spheroidal fruits could be expressed as follows:

(1) $$Sc=f^{2}m^{3/2}$$

where *Sc* is commonly referred to as the stiffness coefficient, f represents the main frequency, and m is the volume mass of fruits [48]. Huang et al. [65] employed acoustic and impact measurements, as well as compression tests, to measure and compare the firmness of tomatoes. During the acoustic and impact measurement, a plastic probe gently tapped the tomato, and the impact sensor recorded the firmness by the probe based on Hertz’s theory; meanwhile, the acoustic sensor obtained acoustic wave signals to calculate the firmness. For the compression test, a metal disc was used to measure firmness by squeezing the tomatoes with low levels of force. The acoustic firmness for tomatoes were distributed between 1.85 and 14.45 (10^4^ Hz^2^g^2/3^, where g is the mass of the fruit in grams), and the impact firmness (dimensionless) was scattering between 7.55 and 75.35. For the compression peak forces of tomatoes, the values ranged from 5.03 to 59.51 N. The results showed that the impact and compression firmness had better correlations with visible and near-infrared spectra, since both spectra and impact firmness as well as compression firmness are locally detected. Similarly, Bart et al. [84] compared the detection performance for two nondestructive firmness sensors based on the acoustic impulse response (AFS) and low-mass impact (SIQ-FT), respectively, for assessing the quality of apples and tomatoes during storage. The acoustic firmness values for tomatoes were distributed between 4 and 10 (10^4^ Hz^2^g^2/3^), while the impact firmness (dimensionless) values ranged from 8 to 16. According to the results, it was found that both devices, especially the SIQ-FT device, showed good accuracy for apples, whereas the detection performance was relatively lower for tomatoes, particularly for the tomatoes that softened over time, which suggests that tomato sorting based on the firmness should be rapidly conducted after post-harvesting to guarantee the detection accuracy. Baltazar et al. [50] proposed an acoustic/impact technique to classify the ripeness of tomatoes, which measured resonant frequency to determine the elastic properties of samples. The frequency generated by impacting tomatoes could be obtained through two acoustic sensors, which were arranged at right angles. The values of acoustic firmness for tomatoes were distributed from 2 to 9 (10^4^ Hz^2^g^2/3^). Data fusion was used to decrease experimental errors to achieve the classification error rate for a tomato ripeness of 9%. However, it is necessary to place tomatoes on a platform made of a specific material to isolate noise and external vibrations, which is a major challenge in practical applications. The previous studies have demonstrated that the acoustic technique is an effective measurement for reflecting global properties of the sample; however, improving the detection performance is still a challenge that limits its application.

In comparison to the aforesaid acoustic methods, the ultrasound technique is capable of measuring deeper internal structures by using higher frequency waves, resulting in the enhanced accuracy of detection. Ultrasound devices are composed of a wave generator (pulser/receiver), a transmitter and receiver, and a computer with signal processing software [85]. When ultrasound devices interact with samples, absorption and scattering make the velocity of the ultrasound signal varied and attenuated. The diversity of internal structures and compositions in fruits and vegetables would lead to discrepant attenuation rates of ultrasound signals, demonstrating unique ultrasound responses for each fruit and vegetable. The ultrasound technique has the capability of monitoring the internal defects, hardness, maturity, and moisture content of fruits during harvesting, storage, and packaging processes [86]. Amos et al. [51] used the ultrasonic technique to assess tomato quality throughout storage. Ultrasonic transmitters and probes were applied to measure the attenuation of ultrasonic waves that passed through the tomato tissue. They discovered that as tomatoes softened during storage, both the ultrasonic signal attenuation and firmness decreased linearly, resulting in the mean value of firmness decreasing from about 8.9 down to 2.2 N. This demonstrated that the ultrasonic technique is viable for tracking the degradation of tomato firmness and quality during shelf life. However, the uneven surface may cause the scattering or reflection of the ultrasound waves, which could observably affect the accuracy and reliability.

### 4.2. Electromagnetic Technology

The development of electromagnetic technology provides a new means for the nondestructive detection of fruits and vegetables [87,88]. Electromagnetic waves are formed by the coupling of electric and magnetic fields, which enable them to propagate in a vacuum and various substances. The commonly used nondestructive detection techniques include visible light (400~750 nm), infrared (750~1000 nm), microwaves (1000~2500 nm), and X-rays.

#### 4.2.1. Machine Vision Technique

**Figure 2:**
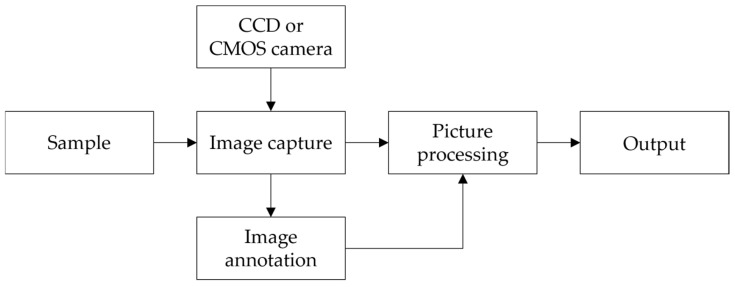
The specific flow chart of machine vision.

The machine vision technique, in brief, employs machines to replace human eyes to achieve measurement and judgment [89]. A CCD or CMOS camera is commonly used in the machine vision technique to capture the surface images of the objects; subsequently, the acquired images are processed, using methods such as feature extraction and image enhancement, and after this, the relationship between the obtained images and quality parameters can be established. The specific flow chart is shown in Figure 2. Such a technique has been widely used in agricultural engineering, since it is capable of inspecting the samples with fast speed, uniformity, and precision [90,91]. Nyalala et al. [53] estimated the weight and volume of single tomato and tomatoes with varying levels of occlusion using machine vision. The average values for the weight and volume of tested tomatoes were 163.18 g and 154.69 cm^3^. After the tomato on the conveyor belt was photographed, image features were extracted to establish seven models (i.e., Linear support vector machines (SVMs), Quadratic SVM, Cubic SVM, RBF SVM, Bayesian regularization artificial neural network (ANN), Levenberg–Marquardt ANN, and Scaled conjugate gradient ANN). The Bayesian regularization artificial neural network outranked all models in weight estimation with a root-mean-square error (RMSE) of 1.468 g and R^2^ of 0.971. For volume estimation, the radial basis function (RBF) SVM had the best performance with an RMSE of 1.2683 cm^3^ and R^2^ of 0.982. It has been proved that machine vision is feasible for tomato online detection. Ropelewska et al. [52] used digital cameras to capture images of tomatoes and converted these images into color channels *R*, *G*, *B*, *L*, *a*, *b*, *X*, *Y*, and *Z*. A correlation analysis was conducted between the texture parameters and the content of tomato lycopene, and the R value was −0.99 from color channels *G*, *b*, and *Y*. The concentration of substances could be determined by measuring their transmittance or absorbance intensity using visible light, which is often applied in conjunction with spectroscopy to assess the quality of agricultural products.

#### 4.2.2. Vis/NIR Spectroscopy

Visible (Vis) spectroscopy, with a spectral region from 400 to 780 nm, is often applied in conjunction with near-infrared (NIR) spectroscopy over the spectral region of 780~2500 nm to assess the quality attributes and safety of the object [92]. Since the spectral region of such a technique corresponds to the absorption bands of overtones and combinations of vibrations in organic molecules that contain hydrogen-containing groups, the characteristic information of organic molecules that contain hydrogen groups for the sample could be obtained by scanning the NIR spectra with the modes of reflectance, transmittance, and interactome. Due to the advantages of no sample preparation, rapid speed, efficiency, and low cost, Vis/NIR spectroscopy is widely used in determining the SSC, pH, moisture content, maturity, and firmness of fruits and vegetables [93,94]. Shao et al. [55] applied the Vis/NIR technique to measure tomato quality characteristics including firmness, SSC, and pH. The reflectance spectra of 200 tomatoes in the range of 350~2500 nm were collected to build partial least squares regression (PLS) models for each quality parameter. According to the results, it was found that the PLS model was more accurate in determining SSC rather than others. Sirisomboon et al. [56] explored the ability of the NIR (1100~1800 nm) technique to classify ripeness and predict the firmness, alcohol insoluble solids, and SSC of tomatoes. The principal component analysis (PCA) model was used to identify the ripeness, while the PLS mode was utilized to predict the quality attributes. The results indicated that the PCA model achieved 100% in ripeness classification. The PLS model could only predict firmness and SSC very well but had a poor performance in predicting alcohol insoluble solids, due to the possibility of the small content in tomatoes. Sun et al. [60] also employed the NIR technique over the spectral range of 950~1650 nm to evaluate SSC in cherry tomatoes. After the Savitzky–Golay (SG) method combined with multiplicative scatter correction (MSC) was utilized to reduce noise, the iteratively retaining informative variables (IRIVs) algorithms were applied to reduce the dimension of the spectral data, so as to improve the support vector regression (SVR) model for SSC prediction, with an R^2^ value of 0.9718. The above studies demonstrated that the NIR technique is an effective measurement technique for tomato quality assessment, but the researchers did not compare the detection performance in different wavelength bands. Huang et al. [95] utilized two spectrometers covering the visible and short-wave near-infrared (Vis/SWNIR) (400~1100 nm) and NIR (900~1700 nm) regions to collect the spectrum of 600 tomatoes at six maturity stages and the firmness of tomatoes was assessed through various tests, including acoustic, impact, compression, and puncture methods. The results showed high prediction accuracy for tomato firmness characteristics like impact firmness, puncture slope, and compression firmness, with Vis/SWNIR spectra outperforming NIR in accuracy. Furthermore, Tiwari et al. [57] used a handheld Vis/NIR spectrometer in the region of 400~1000 nm to investigate the tomato ripeness with an 85% recognition rate in order to help workers identify unripe green tomatoes at harvest, which suggests that the use of handheld spectrometers is a developing tendency, and enhancing the detection performance is significant work. However, Vis/NIR spectroscopy is a point measurement technique with the inability to provide spatial information for the sample, which restricted the application of the quality assessment for tomatoes.

#### 4.2.3. Hyperspectral Imaging Technique

**Figure 3:**
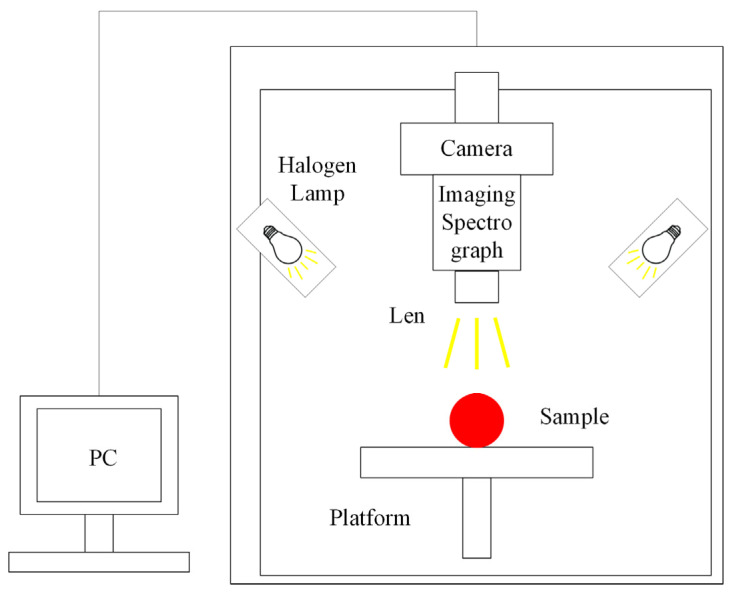
Hyperspectral imaging platform.

Hyperspectral imaging (HSI), combined with spectroscopy and the machine vision technique, has the potential to obtain spatial and spectral information from the samples, which could be used to evaluate the external and internal quality attributes of the objects simultaneously [96,97]. The hyperspectral image is a three-dimensional data cube with one-dimensional spectral information and two-dimensional geometric space, allowing for the acquisition of continuous and narrowband image data with a high resolution [98,99]. The hyperspectral imaging system consists of an imaging module, illumination module, lifting module, and software module (Figure 3).

HSI emerged as a prominent nondestructive technique for the quality assessment of tomatoes. For tomato maturity detection, HSI combined with graph-based semi-supervised methods achieved an impressive accuracy of 96.78%, demonstrating its effectiveness for quality evaluation [68]. Moreover, Dai et al. [70] employed HIS (480~1002 nm) to identify tomato ripeness and predict the lycopene content at different ripening stages. Standard normal variable (SNV) transformations for spectral preprocessing, combined with competitive adaptive weighted sampling (CARS) for selecting feature wavelengths, have been proven to qualitatively recognize the tomato ripeness by a support vector classifier (SVC) with a classification accuracy of 0.958, and to predict lycopene content by SVR and PLS models with correlation coefficients of more than 0.95. Similarly, Zhang et al. [71] employed HSI (400~1000 nm) to establish PLS prediction model for tomato SSC with r value of 0.7925. The results revealed a positive correlation between increased nitrogen levels and the SSC of tomatoes. Furthermore, HSI in the near-infrared region was also applied in tomato quality evaluation. Zhao [100] proposed the near-infrared HSI technique at the spectral range of 980~1660 nm to ascertain the firmness, SSC, lycopene, and pH of tomatoes at three ripeness stages. After HSI images were acquired, Formula (2) was used to correct raw images to minimize the influence of the external environment on image quality.

(2) $$C_{r}=\frac{C_{I}}{2C_{b}}$$

where $C_{r}$ is the corrected reflection image, $C_{I}$ is the original image, and $C_{b}$ is a grayscale reference image combined with 50% black and 50% white images. Tan et al. [69] used the Vis/NIR HSI system, covering 376~1699 nm, to measure the firmness, SSC, and titratable acidity (TA) of cherry tomatoes. Multiple mathematical models (i.e., PCR, PLS, SVM, and Back propagation (BP) Neural Network) were established by using individual spectra and fused spectra, respectively. The results showed that the fused spectra obtained better predictions with R^2^ values of more than 0.85 for each quality parameter. Simultaneously, some studies reported that the wavelength range of 1300 to 1500 nm exhibited the best performance for both maturity classification models and quality prediction models [101]. Despite this progress, challenges remain, like the influence of surface irregularities on spectral data. Moreover, HSI enables rapid and noninvasive assessments of the sample; the initial setup and data processing are complex, which are also the factors limiting its application. Therefore, some studies adopted multispectral imaging to replace hyperspectral imaging, so as to simplify the redundant data. Liu et al. [67] employed multispectral imaging to predict the tomato lycopene and phenolic content by obtaining images at specific bands. PLS, LS-SVM, and BPNN were applied to develop quantitative models. The BPNN achieved the highest R^2^ values of 0.938 and 0.965 for lycopene and phenolic content predictions. Although this method could reduce or simplify the complex data, it is necessary to know the feature bands for quality parameters to acquire excellent results.

#### 4.2.4. Optical Property Measurement Technique

**Figure 4:**
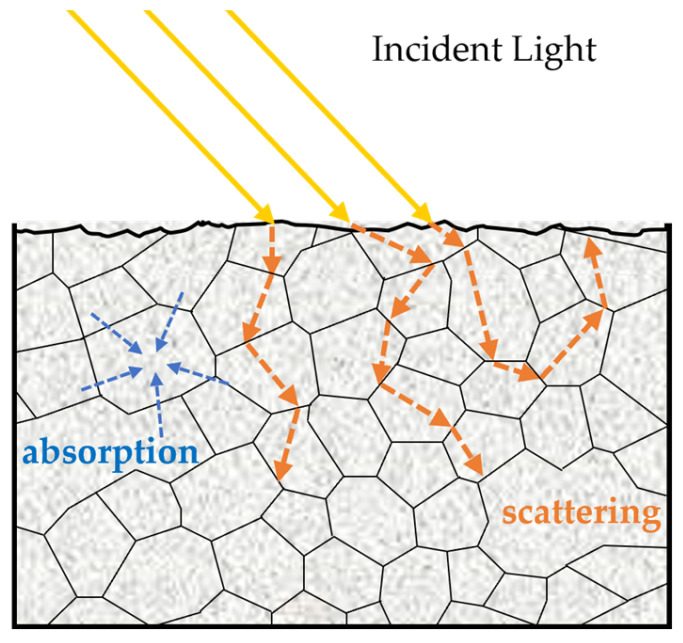
Absorption and scattering between light and biological tissues.

As photons enter biomaterial tissues, scattering and absorption occur, which are, in turn, related to the chemical composition and tissue structure [102], as shown in Figure 4. Absorption involves the annihilation of photons to be converted into different forms of energy, such as heat or chemical energy, while scattering occurs when the light passes the discrete particles or when variation occurs in the refraction index, resulting in changes in light travel direction [103,104]. Conventional Vis/NIR spectroscopy only provides information on the aggregate effect of absorption and scattering, which might lose some detailed information from the samples. Therefore, optical property measurement techniques have attracted attention from researchers. Time-resolved spectroscopy (TRS), frequency domain spectroscopy (FDS), spatially resolved spectroscopy (SRS), spatial frequency domain imaging (SFDI), and integrating sphere (IS) techniques are the mainly used optical property measurement techniques to evaluate quality attributes of fruit and vegetables [105].

The TRS normally utilizes a short pulse of monochromatic light to be injected into the object; whenever a photon strikes scattering particles, it undergoes multiple scattering and the propagation direction changes at each scattering event before it is absorbed or reemitted across the boundary [106]. By measuring the time-resolved reemitted light intensity signals at a certain separation away from the light source along with the recorded temporal response, the absorption coefficient (*μ_a_*) and reduced scattering coefficient (*μ_s_*′) of the tissue are calculated by an inverse algorithm of the diffusion approximation equation. In the FDS system, the light source is modulated at a high frequency to produce steady-state light with varying spatial phases (*α*) and frequencies (*f*). This modulated light is then incident onto the tissue, and the emergent light is measured at a specific distance. Due to the absorption and scattering properties of the medium, the emergent light generates amplitude variations and phase delay. By fitting the observed amplitude variation (*M*) and phase delay (*θ*) at a radial position (*r*) to the light transport model, the *μ_a_* and *μ_s_*′ are then estimated. In contrast to single-probe detection for TRS and FDS techniques, the SRS technique measures signals by placing multiple detection fibers at different spatial locations away from the point light source to acquire spatially resolved diffuse reflectance spectra, from which the optical property parameters like *μ_a_* and *μ_s_*′ can be extracted by an inverse algorithm with an appropriate analytical solution to the diffusion equation. Compared with the TRS and FDS techniques, the SRS technique is distinguished by the simplicity of the instruments, straightforward operation, and relatively wide wavelength coverage. As an emerging optical imaging technique, the SFDI technique is capable of measuring the tissue optical properties in a wide-field area on a pixel-by-pixel basis to achieve a comprehensive analysis. The SFDI technique obtains depth-resolved information about the composition and structure of the object by varying the frequency of modulated light to quantify the optical absorption and approximate scattering coefficients of tissues [107]. In contrast to TRS, FDS, and SRS techniques, the SFDI technique utilizes spatially modulated area lighting instead of point illumination [108]. Thus, it is possible to achieve two-dimensional or three-dimensional optical property mappings through a single measurement. The SFDI technique is widely used for measuring the optical properties of biological materials due to the features of wide-field, label-free imaging and depth-resolved imaging [107].

In recent years, optical property measurement was also employed to inspect tomato quality. Cubeddu et al. [109] employed TRS to assess the internal optical properties of tomatoes. Time-resolved reflectance measurements of tomatoes were carried out from 650~1000 nm to calculate the *μ_a_* and *µ_s_*′. The reflectance *R*(*r*,*t*) could be obtained by the following equation [110]:

(3) $$R\left(r,t\right)=\frac{z_{b}}{(4\pi Dc{)}^{3/2}t^{5/2}}\cdot \exp\left(\frac{r^{2}+z_{b}^{2}}{4Dct}\right)\cdot \exp\left(-\mu_{a}ct\right)$$

(4) $$D={\left[3(\mu_{a}+\mu_{s}\prime )\right]}^{-1}$$

(5) $$z_{b}=1/\left(\mu_{a}+\mu_{s}\prime \right)$$

where D is the diffusion coefficient and *c* is the speed of light in the medium. The results showed that the absorption coefficient spectrum shape was influenced by the chlorophyll *a* content and water absorption, while the scattering coefficients decreased progressively with the increasing wavelength. Despite the fact that TRS is suitable for the optical characterization of agricultural products due to its high measurement accuracy, its application is constrained by several limitations including the high cost of the light source equipment in the system, the high sensitivity requirements of the detector, and the difficulty of the measurement probe in establishing complete contact with the surface of samples [111]. Similar to the HSI, SRS also has capability to provide both spatial and spectral information for the sample with exceptional characteristics of penetration depth information. Huang et al. [66,95,112] extensively explored the application of SRS in the nondestructive detection of tomato quality, focusing on key parameters such as firmness, SSC, pH, and maturity. Due to the different light source-detector distances, each SR spectra contained tomato information at various depths, which presented comprehensive information for the samples. PLS models were employed to establish the relationship between spectral signatures with firmness, SSC, pH, and maturity. The results found that SRS was capable of predicting firmness, SSC, and pH, and identifying tomato maturity stages, demonstrating that SRS is advantageous for assessing the condition and properties of tissues at different depths. Additionally, SRS was applied to obtain the optical properties of tomatoes by the diffusion model and inversion algorithm [113,114]. The analytical equation for spatially resolved diffuse reflectance is shown as follows [115]:

(6) $$\begin{array}{c} R\left(r\right)=C_{1}\phi \left(r,z=0\right)+C_{2}\vec{J}\left(r,z=0\right)\\ \;=\frac{C_{1}}{4\pi D}\left[\frac{\exp\left(-\mu_{eff}r_{1}\right)}{r_{1}}-\frac{\exp\left(-\mu_{eff}r_{2}\right)}{r_{2}}\right]\\ \;+\frac{C_{2}}{4\pi }\left[z_{0}\left(\mu_{eff}+\frac{1}{r_{1}}\right)\frac{\exp\left(-\mu_{eff}r_{1}\right)}{r_{1}^{2}}+\left(z_{0}+2z_{b}\right)\left(\mu_{eff}+\frac{1}{r_{2}}\right)\frac{\exp\left(-\mu_{eff}r_{2}\right)}{r_{2}^{2}}\right] \end{array}$$

(7) $$z_{0}={\left(\mu_{t}^{\prime }\right)}^{-1}={\left(\mu_{a}+\mu_{s}^{\prime }\right)}^{-1}$$

(8) $$\mu_{eff}=\sqrt{3\mu_{a}\left(\mu_{a}+\mu_{s}^{\prime }\right)}$$

(9) $$z_{b}=2AD,\;A=\left(1+R_{f}\right)/\left(1-R_{f}\right)$$

(10) $$\mu_{eff}=\sqrt{3\mu_{a}\left(\mu_{a}+\mu_{s}^{\prime }\right)}$$

(11) $$\mu_{eff}=\sqrt{3\mu_{a}\left(\mu_{a}+\mu_{s}^{\prime }\right)}$$

(12) $$z_{b}=2AD,\;A=\left(1+R_{f}\right)/\left(1-R_{f}\right)$$

(13) $$D={\left(3(\mu_{a}+\mu_{s}^{\prime })\right)}^{-1}$$

(14) $$C_{1}=\frac{1}{4\pi }\int_{2\pi }\left[1-R_{fres}\left(\theta \right)\cos\theta d\omega \right]R(r),\;C_{2}=\frac{3}{4\pi }\int_{2\pi }\left[1-R_{fres}\left(\theta \right)\cos^{2}\theta d\omega \right]$$

where $r_{1}$ is the distance from the detector to the actual light source, *r*^2^ is the distance from the detector to the mirror light source, $\mu_{eff}$ is the attenuation coefficient, $z_{b}$ is the reflection coefficient inside the tissues, D is the diffusion coefficient, and $C_{1}$ and $C_{2}$ are the coefficients produced by the refractive index. The refractive index n is usually 1.35 for fruits and vegetables, while $C_{1}$ and $C_{2}$ are 0.1277 and 0.3269 [115]. The results showed that the shapes of the absorption coefficient and scattering coefficient spectra for tomatoes are similar to the study using TRS [69]. Moreover, the *μ_a_*, *µ_s_*′ and their combinations were used to establish PLS models to predict the firmness, SSC, and pH of tomatoes. However, since the values of *μ_a_* and *µ_s_*′ are too small, errors are easily introduced, leading to relatively lower results. In addition, a potential drawback of SRS is that there are no specific criteria for the selection of light sources. The utilization of a high-power light source had the possibility to irreversibly damage both external and internal biological tissue. Further, a low-power light source is not capable of acquiring adequate information from the sample, resulting in unsatisfying detection performance. Consequently, further research is required to facilitate its application [116]. Currently, SFDI as an emerging technique normally used in the field of measuring early bruised fruit. Sun et al. [107] designed a multispectral SFDI system to detect early-stage bruising in tomatoes. They found that the reduced scattering coefficient mapping could detect early-stage bruising, and effective characteristic wavelengths could improve the accuracy of bruise detection. The above studies demonstrated that optical property measurements have potential in the field of quality assessment for tomatoes. However, the values of the optical parameters are too small, which means that errors are easily introduced; thus, guaranteeing the values of these optical parameters accurately is a significant challenge.

#### 4.2.5. Raman Spectroscopy

Raman spectroscopy acquires molecular vibration information through frequency shifts and intensity changes in scattered light when the laser light source interacts with samples; this technique is used to analyze and identify the chemical composition, structure, and properties of the samples [117,118]. As the advancement of the electromagnetic field and chemical effects are enhanced, the intensity of the Raman spectra improved, which promotes surface-enhanced Raman spectroscopy beyond the constraints of low detection sensitivity for conventional techniques [119]. Since the Raman scattering of water is weak, Raman spectroscopy is an ideal means to inspect biological samples and chemical compounds in aqueous solutions. Additionally, Raman spectroscopy addresses limitations in chemical specificity compared to HSI and SRS, which means that such a technique excels at measuring harmful substances and surface pesticide residues in fruits and vegetables [120]. Fu et al. [74] employed a miniaturized Raman spectroscopic system alongside colorimetric analysis to assess the tomato lycopene content. They leveraged the sensitivity of Raman spectroscopy to molecular vibrations to address the lycopene content, and the results indicated that this system provides rapid and reliable measurement for tomato quality evaluation. However, the irregular surface of tomatoes may potentially affect the Raman signal quality. Tao et al. [121] used surface-enhanced Raman spectroscopy (SERS) to measure pesticide residues on the surface of tomatoes. The flexible tape was firstly pasted on the tomato surface for sampling, and the Raman spectrum from the tape was acquired for data analysis. According to the results, it was found that this method could be applied to the online semiquantitative nondestructive detection for different kinds of pesticides. Qin et al. [73] explored spatially offset Raman spectroscopy (SORS) for the nondestructive evaluation of tomato maturity. They developed a Raman system using a 785 nm laser to collect the spatially offset spectrum in the wavelength range of 200~2500 cm^−1^. The pigment content of tomatoes at different maturity stages was different, leading to various Raman spectrum peaks. Pure lycopene was used as a reference, and the variation in Raman peaks was recorded by spectral information divergence (SID) to evaluate the maturity of tomatoes. These studies suggest that Raman spectroscopy has potential in the application of tomato quality assessment.

#### 4.2.6. X-Ray Technique

**Figure 5:**
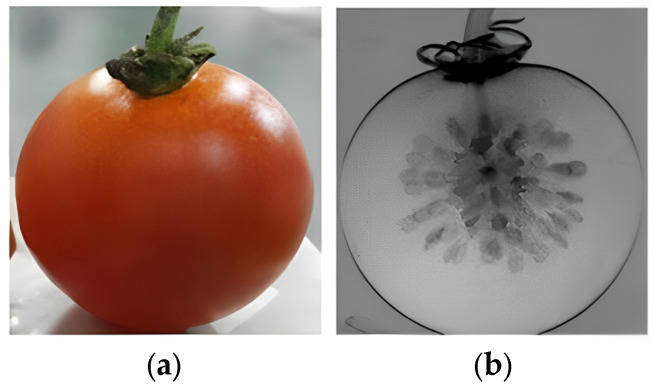
A fresh cherry tomato (**a**) and its scattering image (**b**).

X-ray, electromagnetic radiation, is emitted by electrons outside of the atomic nucleus with the characteristic of short wavelengths (about 0.02 to 10 nm) and high energy (0.12~120 keV) to achieve good penetration ability [122]. In this paper, X-ray imaging (XRI) and X-ray computed tomography (CT) are grouped into X-ray techniques. The principle of X-ray techniques is based on the differential absorption of X-rays across different tissues [123]. X-ray imaging (XRI) enables the acquisition of variable grayscale intensity images that are a two-dimensional (2D) projection of a three-dimensional (3D) object. X-ray CT is capable of acquiring images from multiple angles coupled with mathematical algorithms to reconstruct 3D images of the object [124]. Wang et al. [75] proposed X-ray Talbot–Lau interferometry to image fresh cherry tomatoes and dried plums. Figure 5 shows photographic images of fresh cherry tomatoes and X-ray images based on grating, which revealed abundant information about the surface and interior of tomatoes and suggested a high potential for X-ray techniques in nondestructive detection for tomato quality evaluation. However, grating-based X-ray imaging is time-consuming. Further upgrades are needed in grating interference scanning systems with multiple line detectors and manipulation mechanisms, which may promote the application of X-ray imaging. Moreover, X-ray techniques are limited in the application of tomato industries for quality assessment and safety due to the high cost of instruments and the long time for 3D reconstruction [125].

#### 4.2.7. Nuclear Magnetic Resonance Technique

The nuclear magnetic resonance (NMR) technique employs the magnetic properties of atomic nuclei and their interaction with radio frequencies as well as applied magnetic fields to achieve magnetic resonance imaging (MRI) that is capable of visualizing the spatial distribution of protons in the sample [126]. The NMR technique is good at analyzing hydrogen nuclei due to their spin properties in magnetic fields to generate magnetic moments. In addition, NMR is characterized by strong penetration, high resolution, good repeatability, and not being limited by the shapes of samples. Thus, it is mainly employed to detect the water content and moisture distribution in fruits and vegetables [127]. Zhang et al. [128] applied the MRI technique to analyze the red layer in tomato skin in three tomato varieties. The permanent magnet MRI system was performed to image tomatoes and each image was sliced longitudinally. The red layer and peel tissue were characterized based on morphological features and the signal intensity of magnetic resonance imaging. The differences in the red layer in three varieties were compared and analyzed, resulting in percentages of 56.9%, 50.9%, and 30.4%, respectively. The results showed that the separability of tomatoes was correlated with the features of the red layer and peel. However, the number of varieties in the experiments was relatively small, and further research could validate the results across more varieties and explore the quantitative relationship between peel characteristics as measured by MRI and separability. The MRI technique is also applied in qualitative analysis for tomatoes. Sequi et al. [129] employed the MRI technique to obtain a profile image of fresh cherry tomatoes to measure physical (i.e., transverse relaxation times) and morphological (i.e., pericarp thickness and sample diameter) parameters, so as to determine the geographical origin and cultivated variety. According to the results, it was found that this method is capable of distinguishing the geographical origin for fresh cherry tomatoes with a recognition rate of 80% and identifying the cultivated variety with a classification accuracy of 90%. Borba et al. [76] used time-domain nuclear magnetic resonance (TD-NMR) to detect the relaxation signal of tomatoes in order to develop classification models for tomato ripeness (i.e., green, unripe; red, ripe), SSC (i.e., °Brix ≤ 4.5 or °Brix > 4.5) and defects. The relaxation signal was related to the fluidity of water in the tissue; the attenuation signal of the green tomato was shorter than that of the red tomato. According to the results, it was found that TD-NMR had the capability to identify tomato ripeness based on the SVM model with a classification accuracy of 97%, to recognize tomato SSC based on SVM and K-nearest neighbor (KNN) models, both of which had recognition rates of 100%, and to inspect the tomato defects based on the PLS model, with an accuracy of 90%. However, long imaging times, expensive equipment, a high magnetic field, and the higher energy required yielding a large carbon footprint, restrict the application of the NMR technique in the food industry and agricultural engineering [130].

### 4.3. Electrochemical Sensor Technology

**Figure 6:**
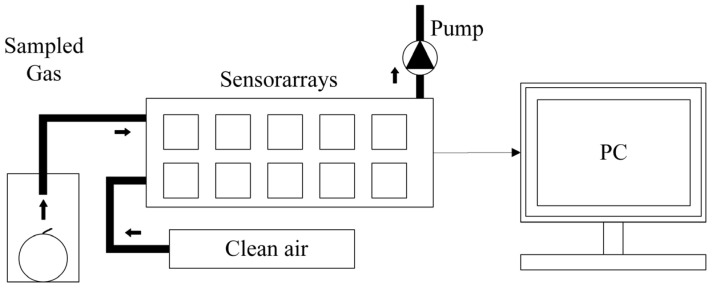
Schematic diagram of the electronic nose measurements.

Electrochemical sensors, including electronic noses and electronic tongues, are based on the electrochemical properties of samples, which convert chemical quantities into electrical quantities for nondestructive detection. The electronic nose technique is typically composed of an electronic chemical detector unit containing an array of sensors and appropriate pattern recognition systems to respond to gases and volatile compounds emitted from samples. The measurement process is illustrated in Figure 6; the gases emanating from the sample are piped into the sensors. During the measurement, the system takes samples at intervals of one second. The gas emitted by the measured sample interacts with the active material of the sensor arrays, resulting in an instantaneous response converted into an electrical signal.

The electronic tongue is defined as a multisensory system for liquid analysis based on chemical sensors that simulate the organization of human taste buds coupled with pattern recognition methods [131]. The principle of the electronic tongue is that the sensory array is stimulated by the sample to induce and generate signals into the computer; the results of electronic tongue are analyzed through signal procession and mathematical computation. Typically, electronic tongues are more sensitive to samples containing a higher concentration of polar compounds [132].

Compared with the aforementioned techniques, electrochemical sensors offer several advantages, including low power consumption, high sensitivity, and high resolution. Gómez et al. [77] employed a bespoke electronic nose device comprising 10 different metal oxide sensors to distinguish different ripeness states for tomatoes. Samples were placed in sealed containers, and the odor of samples was pumped to the sensors of the electronic nose for detection using an air pump. PCA and linear discriminant analysis (LDA) were used in the data analysis. The results indicated that the electronic nose was capable of distinguishing the ripeness states of tomatoes and LDA enabled the detection of significant differences in the volatile characteristics of tomatoes with a recognition rate of 100% compared with PCA, which had a 95.79% classification accuracy. The electronic nose technique is not only used in qualitative analysis for tomatoes but also for quantitative analysis. Zhou et al. [133] developed a portable electronic nose system for tomato storage quality prediction based on several mathematical models (i.e., KNN model; Decision Tree (DT) model; SVM model; SVM combined with loop optimization algorithm (LOA-SVM) model; SVM model characterized by whale optimization algorithm (WOA-SVM)). The gas signals for the electronic nose were collected from the tomatoes at different storage times over 21 days. Subsequently, the mildew degree of tomatoes was observed visually, which corresponded to the gas signal of tomatoes. The result found that the WOA-SVM model presented the best prediction, with a 99.81% accuracy compared with other models, which indicated that the WOA-SVM model had the advantages of fast analysis, good repeatability, and high accuracy. The mechanism of the electronic tongue was analogous to that of the electronic nose. Xu et al. [134] used the electronic tongue to differentiate the taste characteristics of refrigerated or blanched tomatoes, incorporating different varieties and different ripeness levels. However, in contrast to the electronic nose, the electronic tongue requires tomato juice for detection; therefore, it is tough to guarantee the integrity of samples by using the electronic tongue. Furthermore, the application of the electronic tongue and electronic nose is somewhat constrained by a number of factors, including the materials used in the sensors, the manufacturing processes employed, and the methods used for data analysis.

### 4.4. Technique Comparison

The nondestructive detection technique has become a crucial means for the assessment and analysis of tomato quality. Since each of these means is designed to meet specific objectives and requirements, they also exhibit distinct advantages and limitations, which bring challenges to their widespread adoption in the food industry. Table 2 presents the comparison of characteristics for nondestructive detection techniques in terms of tomato quality assessment.

Mechanical characteristic techniques, including mechanical loading, impact, acoustic, and ultrasonic testing, are great at evaluating mechanical properties. Mechanical loading can directly measure the mechanical properties of the fruit without the external environment’s effects like noise and stray light, but the results would be affected by the uneven surface of fruit. The impact method is simple to operate and relatively cheap, but it only measures local firmness. Acoustic and ultrasonic waves could reflect the overall firmness and are suitable for fruit with irregular shapes or uneven sizes [135]. Moreover, ultrasonic waves have high sensitivity to the change in tissue density, lacuna, and water content in fruit [86]. However, their detection accuracy is influenced by ambient noise or vibrations during signal acquisition in the practical application [136] and ultrasonic waves need to be calibrated for varying acoustic characteristics of different fruits [137]. Electromagnetic techniques allow for the noninvasive detection of kinds of physical and chemical quality parameters in tomatoes. Spectroscopy techniques, offering spatial and/or spectral information, provide comprehensive chemical and physical analysis, especially Raman spectroscopy, which delivers highly sensitive molecular composition analysis though it is limited by data complexity or slow detection speed [120]. Vis/NIR spectroscopy is widely used in the field of quality assessment for fruit and vegetables due to the advantages of simple operation, no sample pretreatment, and relatively cheap devices, while the point detection and lack of spatial information limit the practical application [66]. Recently, HSI, with the superiority of providing spatial and spectral information simultaneously, has become more and more popular in quality evaluation for fruit [138], but the HSI instrument is expensive. Compared with HSI, spatially resolved spectroscopy as an emerging technique also offers spatial and spectral information together with depth information; such a technique enables the analysis of samples with independent spectra or the exploration of sample optical properties by using combination spectra. The optical property method has developed rapidly in the fruit industry, since it can provide optical absorption and scattering information separately, which is significant for analyzing light transmission in the sample tissue [139]. However, the obtained values for optical absorption and scattering coefficients are relatively small, which make them easy to introduce errors, leading to lower accuracy for detection performance [45]. X-ray imaging [140] and time-domain NMR [76] are highly effective for internal defect detection and component quantification; however, the high-cost devices and slower detection speed limit their applications in tomato analysis [141]. Electronic noses and tongues are inexpensive and sensitive for measuring volatile compounds and liquid samples with fast detection speed [142]. However, such techniques are influenced by environmental factors and sensor limitations [80]. In addition, compared with the electronic nose, the electronic tongue is suitable for measuring liquid samples due to the requirement of juice for detection.

The application for each technique depends on specific needs and trade-offs. In future research, it is possible to use a combination of different techniques that will enable the detection of more tomato quality parameters with a simpler and cheaper method.

## 5. Deep Learning with Nondestructive Detection Technology in Application of Tomato Quality Assessment

Nondestructive detection techniques make tomato quality grading more effective and efficient since they can obtain comprehensive information from the sample. However, as the information or data increase, conventional machine learning methods are not able to process effectively and give robust results, which limits the application of the nondestructive technique. As artificial intelligence developed, deep learning was proposed and applied in various fields, which brought new hope for nondestructive techniques to evaluate quality attributes [143,144,145].

Deep learning, a subset of machine learning, represents a novel direction within machine learning, which has excellent outcomes combined with nondestructive techniques in various areas. Deep learning is normally based on artificial neural networks, which automatically extract and learn feature patterns from data through multiple layers of nonlinear processing units [146]. Commonly used deep learning algorithms include convolutional neural networks (CNNs), Recurrent Neural Networks (RNNs), Generative Adversarial Networks (GANs), autoencoders, and Deep Belief Networks (DBNs). CNNs are a prevalent tool in image processing, which extract local features by employing convolutional operations to render them particularly well-suited for tasks like image classification and object detection [147,148]. RNNs are adept at processing sequential data and are extensively applied in natural language processing and time-series predictions. GANs consist of a generator and a discriminator, both of which are competing against each other to generate high-quality data, with applications ranging from image to text generation [149]. Autoencoders are unsupervised learning algorithms that compress input data into low-dimensional representations and reconstruct them, which are commonly used for tasks such as dimensionality reduction and denoising [150]. DBNs employ layer-wise unsupervised training for feature learning. Such an algorithm enables researchers to capture the hierarchical structure of data during the training process to make them useful for feature extraction and classification tasks [151]. These deep learning algorithms demonstrate powerful learning capabilities across diverse application domains. Recently, deep learning has also been increasingly applied in the nondestructive detection of tomatoes for quality assessment.

### 5.1. Deep Learning in Electromagnetic Technology

#### 5.1.1. Deep Learning in Machine Vision

With the rapid advancement of information science, computer vision-based pattern recognition techniques and image processing methods have become mature and are capable of the applications of quality and safety assessment for agricultural products [152]. Deep learning, coupled with computer vision techniques, promotes quality sorting or grading for the object in a more accurate and effective way, due to its exceptional data processing performance [153,154,155]. Da Costa et al. [156] proposed machine vision technique with a deep neural network binary classifier to identify external defects in tomatoes, which was trained and validated with 43,843 images captured by a tomato sorting machine from a commercial organization in Brazil. The network was constructed using transfer learning, and the optimization was achieved through feature extraction and fine-tuning means. In the feature extraction phase, all weights were locked except for the softmax classification layer, while the fine-tuning method allowed for the partial retraining of the network, refining certain intermediate layers to improve performance for the specific task. The final classification results demonstrated an accuracy of 94.6%. Notably, the model did not rely on manually designed features or prior knowledge of tomato characteristics, making it applicable to other types of agricultural products. Additionally, machine vision combined with deep learning could be employed in tomato harvesting robots [157]. Compared to conventional methods like SVM and PLS, deep learning, particularly for CNNs, emphasizes deeper model architectures and leverages large datasets to learn features. In the previous study, a convolutional transformer combined with convolutional neural networks was proposed for tomato ripeness classification [158]. The framework comprised three principal blocks, i.e., the transformer, the encoder, and the decoder. The input scan was initially passed to the transformer and encoder blocks. At the convolutional transformer side, the input scan was segmented into a set of image blocks from which the position embedding was calculated to autonomously recognize and grade tomatoes. Moreover, the decoder blocks were designed to accurately segment and grade tomato ripening levels by removing extraneous elements through rescaling and maximum non-pooling operations. This deep learning model aimed to handle tomatoes with varying levels of occlusion, different lighting conditions, and ripeness stages. It provided a promising solution for efficiently harvesting tomatoes in complex and diverse natural environments. Liu et al. [159] proposed an enhanced five-layer DenseNet network structure. In this network structure, adjacent unit modules were connected to the average pooling layer through a convolutional layer, which could obviate the necessity for a fully connected layer. This design reduced the number of parameters in the entire network to suppress the phenomenon of overfitting. A structured sparse operation was introduced to divide the network’s convolutional kernels into multiple groups, gradually reducing unimportant parameter connections during the training. Moreover, a focal loss function was implemented in the final classification layer to significantly enhance the model’s generalization capability. Under identical hardware and software configurations, this approach reduced computational complexity by 18% compared to the original DenseNet algorithm.

#### 5.1.2. Deep Learning in Spectral Technique

Spectroscopy has been widely employed in the assessment of internal and external quality in tomatoes. Various linear and nonlinear chemometric methods have been devised for the analysis of spectral data [160,161,162]. The applicability of existing chemometric methods is constrained by the presence of noise in the data. Deep learning approaches were developed to address this issue by reducing spectral noise, extracting features, and performing calibration regression models. The recent advancements in deep learning models suggest that deep learning models can uncover complex structures in large datasets and extract features without the need for manual intervention [163]. Therefore, deep learning coupled with spectroscopic techniques has been increasingly applied in spectral analysis in recent years. The near-infrared hyperspectral imaging technique (980~1660 nm) was employed to assess the firmness, SSC, lycopene content, and pH levels of tomatoes at three maturity stages [100]. Models for predicting four quality parameters and identifying maturity stages were developed using random forest (RF), PLS, and RNN models. The results demonstrated that the classification accuracy for the RNN model was 40% higher than the RF model and 17% higher than the PLS model. Additionally, in predicting quality parameters, the RNN model achieved the highest R^2^ values (>0.87), outperforming both PLS and RF models. This is because deep learning models can automatically learn features from a large number of inputs, allowing the RNN to extract features more accurately to improve the prediction results compared with machine learning models. The combination of near-infrared hyperspectral imaging with deep learning proved to be an effective approach for predicting the quality and maturity of tomatoes. Furthermore, a deep learning regression model based on hyperspectral images was also developed to analyze sugar content in tomatoes [164]. The 1D convolutional ResNet network (Con1dResNet) was comprised of five principal blocks. The initial block consisted of a 1D convolutional layer and a maximum pooling layer. The second block contained three residuals module. The third block contained a downsampled module and three residual modules. The fourth block passed through a downsampled module and five residual modules before the dropout layer with a parameter of 0.5 and then continued through three residual modules. The fifth block consisted of an averaging pool layer and a linear output layer. The Con1dResNet was capable of effectively extracting rich data features. The residual learning structure could facilitate an enhancement in overall performance. In addition, this model was shown to be insensitive to anomalous data. It could be trained using pre-trained models, thus reducing the training cost. The experimental results demonstrated that Con1dResNet could significantly outperform existing machine learning methods, with an R^2^ of 0.901 and an MSE of 0.018. Another exploration combined Raman spectroscopy with CNNs to identify tomato quality [165]. In this study, Raman spectral information of tomatoes was analyzed and trained using a neural network with deep network structure. The deep network structure was mainly divided into three parts, including the input layer, output layer, and convolution layer, and the total number of layers was 47. The concept of factorization was introduced into convolutions of a smaller scale by dividing a larger 2D convolution into two smaller 1D convolutions. This approach had twofold benefits. Firstly, the number of parameters was reduced, thereby enhancing computational efficiency and preventing overfitting. Secondly, a layer of nonlinearity was applied, extending the model’s expressiveness. Consequently, the network was capable of handling a greater number of spatial features and increasing the diversity of features. The results demonstrated that the feature recognition accuracy of the test set could reach 94.6%, which indicates that the tomato quality recognition method based on Raman spectra combined with convolutional neural networks is simpler and faster.

#### 5.1.3. Deep Learning in X-Ray Technique

X-ray CT with advanced imaging capabilities has developed rapidly, extending its applications from medical examinations to other areas of biological detection. In recent years, with the powerful ability for deep feature analysis and the convenience of end-to-end prediction, many researchers have successfully applied deep learning-based segmentation algorithms to CT image processing, achieving outstanding results [166]. X-ray imaging relies on differential X-ray attenuation across various tissues, allowing for the revelation of internal seed morphology. A large amount of data from X-ray images must be analyzed in order to assess the physiological quality of tomato seeds using X-ray imaging. Machine learning methods are often used to interpret image analysis data, with deep learning strategies such as CNNs representing a prominent trend in the field of artificial intelligence. Studies based on the Mask region convolutional neural network (MaskRCNN) have demonstrated a strong capacity for recognizing targets. Pessoa et al. [167] reported on a convolutional neural network known as MaskRCNN to classify tomato seeds into four quality categories with an accuracy of 83.64%. The advantage of the MaskRCNN method over manual approaches is the reduced subjectivity.

Table 3 introduces the recent applications for the combination of nondestructive detection techniques and deep learning for the quality assessment of tomatoes. Vision-based methods, including machine vision and tactile sensing, effectively classify ripeness, firmness, and quality grades, using mathematical models like YOLO, R-CNN, and MobileNetV3. Vis/NIR spectroscopy reliably predicts ripeness and geographical origin with high accuracies using models such as the FCNN and SAE-SSA-SVM. Hyperspectral imaging demonstrates strong predictive power for physicochemical attributes like SSC, firmness, and lycopene content, by advanced models like the RNNs, with R^2^ values of 0.94 for lycopene content prediction and 0.92 for firmness prediction. Raman spectroscopy also shows potential for tomato quality assessment, with CNN models reaching an R^2^ value of 0.946, highlighting sensitivity to chemical composition. These techniques and methods collectively promote precision and efficiency in tomato quality evaluation.

## 6. Limitations and Future Developments

The summaries of previous research in this review focus on the quality evaluation of tomatoes by nondestructive detection techniques, which demonstrate that such techniques are helpful for tomato grading to make it more efficient and accurate. However, many restrictions and limitations still exist for tomato quality assessment by these techniques. The non-uniformity of tomato tissue, i.e., the presence of bubbles or voids in the internal structure, is a potential factor hindering accurate measurements. Additionally, tomatoes have stains and dryness on the surface after harvest, which will affect the detection accuracy. Moreover, external disturbances and instrument responses during signal acquisition are also challenges, and include stray light, light source stability, instrument temperature, and so on. In addition, in large-scale online or real-time detection, the tomato is usually placed on a conveyor belt. Thus, the material of the conveyor belt is also an important factor to be considered, which should be easily distinguishable from the sample when thresholding and also without destroying tomatoes. Furthermore, the cost of expensive instruments is still a significant factor limiting the application of tomato quality assessment. Therefore, there are still many problems to be considered when employing these nondestructive techniques to evaluate tomato quality. Additionally, laser-induced breakdown spectroscopy and terahertz radiation spectroscopy have the potential to be applied in tomato analysis, since they show good capabilities in the detection of other fruits and vegetables [178,179,180,181,182,183,184,185].

Additionally, the data analysis methods and mathematical models used may not always be suitable, resulting in unsatisfying results. Thus, more advanced algorithms, such as deep learning, are expected to improve the detection performance. Recent advancements in deep learning algorithms have shown superior predictive and classification accuracy compared to conventional machine learning methods in data processing. Deep learning has the capacity to extract high-dimensional features from data automatically without manual operation. Recently, such algorithms are also utilized in agricultural engineering for quality and safety evaluation and are expected to drive significant progress in the nondestructive detection of agricultural products. While deep learning algorithms demonstrate remarkable capabilities in numerous applications, there are also notable limitations. Firstly, deep learning models necessitate substantial quantities of labeled data for effective training; in other words, they are challenging to implement in data-scarce domains. Secondly, these models exhibit a considerable demand for computational resources, particularly when the network layers are deep or comprise a large number of parameters. Furthermore, deep learning models are susceptible to overfitting, particularly when there is an inadequate quantity of data, or the model is excessively complex. In such cases, methods such as regularization and data augmentation are essential to enhance the model’s generalization capabilities.

The multi-information fusion techniques are a developed trend. Deep learning is increasingly being sought after in the quality evaluation of agricultural products with nondestructive techniques to improve the classification or prediction results. Currently, smart agriculture, unmanned farms, agricultural Internet of Things (IoT), and agricultural big data platforms are closely linked to AI in enhancing the quality inspection of agricultural products, which presents a new expectation of the integration of deep learning and AI in nondestructive detection to address the challenges for improving quality evaluation performance.

## 7. Conclusions

Since the early 20th century, studies on nondestructive detection technology for the quality assessment and safety of agricultural products have been developed rapidly. This paper outlined the various nondestructive techniques employed in tomato analysis, emphasizing the integration of deep learning in these techniques. Mechanical nondestructive detection techniques are usually used to assess the elasticity and firmness of tomatoes. Quasi-static compression and impact tests are able to determine the local firmness of tomatoes, while acoustic measurement reflects the overall firmness. Ultrasonic techniques offer higher detection accuracy compared to acoustic techniques. Electromagnetic techniques are capable of analyzing a wide range of tomato quality attributes. Machine vision is mainly used for tomato surface quality inspection. Spectroscopic techniques, including Vis/NIR spectroscopy, hyperspectral imaging, optical property measurement, and Raman spectroscopy, are capable of detecting physical and chemical indicators such as moisture, sugar, firmness, and lycopene in tomatoes. The X-ray and NMR techniques have a high detection accuracy for the moisture content and internal defects of tomatoes. Electrochemical sensor techniques, such as electronic noses and tongues, are commonly used to detect tomato flavor, ripeness, and storage quality. However, these techniques still face certain challenges in practical applications, like the cost and environmental effects. In terms of data analysis, deep learning algorithms are predominantly focused on the applications of tomato analysis by spectroscopic techniques. This is possibly because spectroscopic techniques are capable of measuring various tomato quality parameters, thereby generating large volumes of data for training and testing.

Recently, nondestructive detection techniques have demonstrated considerable potential in the applications of agricultural engineering and the food industry. However, the commercial application of nondestructive detection has progressed slowly due to the heterogeneous tissues in agricultural products, the high costs of equipment, and universally inapplicable predictive models. Future research about the nondestructive detection of tomatoes and agricultural products should address several factors, such as instrument versatility for the capability of measuring more quality parameters, the cost of instruments, especially for portable devices, and the combination of deep learning with artificial intelligence for mathematical models.

## Figures and Tables

**Table 1:** Summaries of nondestructive detection applied for quality evaluation of tomatoes.

Technique	Index	Model	RMSEP	Result	Year	References
Mechanical sensor	Firmness	PLS		r^2^ = 0.912	1996	[48]
Nondestructive impact	maturity	CA	0.6448	Acc = 0.823	2009	[49]
Acoustic impact	Ripening	Bayesian classifier		Acc = 0.89	2008	[50]
Ultrasonic	Firmness			R^2^ = 0.916	2007	[51]
Machine vision	Grading	SVM		Acc = 0.9774	2020	[52]
	Weight	Bayesian regularization network	1.468	R^2^ = 0.971	2021	[53]
	Volume		1.2683	R^2^ = 0.982		
Vis/NIR spectroscopy	TSC	(1000~2500 nm)	0.4157	r = 0.9998	2005	[54]
	SSC		0.6333	r = 0.9996		
	Lycopene		21.5779	r = 0.9996		
	β-carotene		0.7455	r = 0.9981		
Vis/NIR spectroscopy	Firmness SSC pH	PLS (350~2500 nm)	1.48	r = 0.81	2007	[55]
			0.16	r = 0.91		
			0.09	r = 0.85		
		PCR	1.43	r = 0.82		
			0.19	r = 0.86		
			0.09	r = 0.83		
	Maturity	PLS (1100~1800 nm)		Acc = 0.9685	2012	[56]
	Firmness			r^2^ = 0.90		
	Maturity	Bayesian classifier		Acc = 0.85	2013	[57]
	SSC	PLS (400~2500 nm)	0.65	r^2^ = 0.75	2015	[58]
	Total acidity		0.06	r^2^ = 0.69		
	Ripeness	PLS (550~750 nm)	0.18	Acc = 0.9067	2017	[59]
	SSC	IRIV-CS-SVR (950~1650 nm)	0.1707	r^2^ = 0.9718	2018	[60]
	SSC	PLS (902~2094 nm)	0.14	r^2^ = 0.92	2019	[61]
	Acids		0.31	r^2^ = 0.88		
	SSC	EPO (780~2500 nm)	0.292	r = 0.8988	2019	[62]
	lycopene		7.45	r = 0.8023		
	SSC	PLS (840~1050 nm)	0.3227	R^2^ = 0.6665	2021	[63]
	SSC	PLS (500~1400 nm)	0.316	R = 0.830	2021	[64]
Spatially resolved spectroscopy (550~1650 nm)	Firmness	PLS	0.52	r = 0.948	2018	[65]
	SSC	PLS	0.38	r = 0.809	2018	[66]
	pH		0.11	r = 0.819		
Multispectral imaging (405~970 nm)	Lycopene Total phenolics	PLS	6.502	R^2^ = 0.501	2015	[67]
			2.329	R^2^ = 0.343		
		LS-SVM	2.602	R^2^ = 0.910		
			0.865	R^2^ = 0.921		
		BPNN	2.292	R^2^ = 0.938		
			0.308	R^2^ = 0.965		
Hyperspectral imaging	Moisture pH SSC	PLS (1000~1550 nm)	0.63	r = 0.81	2017	[44]
			0.06	r = 0.69		
			0.33	r = 0.74		
	Maturity	CSR (400~2500 nm)		Acc = 0.9678	2021	[68]
	Firmness SSC TA	PCR (950~1650 nm)	0.847	R^2^ = 0.736	2023	[69]
			0.279	R^2^ = 0.813		
			0.079	R^2^ = 0.84		
		PLSR	0.783	R^2^ = 0.785		
			0.258	R^2^ = 0.864		
			0.081	R^2^ = 0.817		
		SVR	0.647	R^2^ = 0.862		
			0.234	R^2^ = 0.917		
			0.077	R^2^ = 0.874		
		BP	0.709	R^2^ = 0.816		
			0.193	R^2^ = 0.938		
			0.069	R^2^ = 0.919		
Hyperspectral imaging	Maturity	SVC (480~1002 nm)		Acc = 0.9583	2023	[70]
	Lycopene	SVR	0.0166	R^2^ = 0.9652		
		PLSR	7.9826	R^2^ = 0.9589		
	SSC	CNN (400~1000 nm)	0.4029	r = 0.7932	2024	[71]
		RCNN	0.4199	r = 0.7025		
		GCNN	0.4125	r = 0.7651		
		PCNN	0.4129	r = 0.7977		
Optical properties (550~1300 nm)	Firmness	PLS	0.62	r = 0.923	2018	[45]
	SSC		0.50	r = 0.623		
	pH		0.12	r = 0.769		
Raman spectroscopic (3703 nm)	Lycopene	PLS		R^2^ = 0.91	2006	[72]
	Maturity			Acc = 0.938	2012	[73]
	Freshness	PLSR		Acc = 0.856	2016	[74]
	Lycopene		14.2	r = 0.57		
X-ray	Internal structure				2016	[75]
Time-domain NMR	Maturity SSC	SIMCA		Acc = 0.88	2021	[76]
				Acc = 0.87		
		PLS-DA		Acc = 0.85		
				Acc = 0.90		
		SVM		Acc = 0.97		
				Acc = 1.00		
		KNN		Acc = 0.94		
				Acc = 1.00		
Electronic nose	Maturity	PCA		Acc = 0.9579	2006	[77]
		LDA		Acc = 1.00		
	SSC pH	PCR	0.136	R^2^ = 0.877	2022	[78]
			0.184	R^2^ = 0.748		
		PLS	0.085	R^2^ = 0.865		
			0.185	R^2^ = 0.747		
		SVR	0.345	R^2^ = 0.958		
			0.134	R^2^ = 0.877		
	Ripeness	DCNN		Acc = 0.8220	2024	[79]
Electronic nose and tongue	Freshness	LVQ		E-nose Acc = 0.86 Acc = 0.968	2015	[80]
		Lib-SVM		E-tongue Acc = 0.9688 Acc = 0.9816		

Acc, Accuracy. PLS, Partial least squares regression. CA, Cluster analysis. SVM, Support vector machine. PCR, Principal component regression. EPO, External parameter orthogonal. IRIV-CS-SVR, Iteratively reserved information variables-Cuckoo search-Support vector regression. SIMCA, Soft Independent Modeling by Class Analogy. PLS-DA, Discriminant analysis by partial least squares classification. LDA, Linear discriminant analysis. LVQ, Learning vector quantization. Lib-SVM, library support vector machines.

**Table 2:** Comparison of characteristics for different nondestructive detection techniques in quality evaluation of tomatoes.

Classification	Technique	Advantages	Drawbacks
Mechanical characteristic technology	Mechanical loading	Directly measure the mechanical properties of the fruit	Risk of damaging fruit
		Fast detection speed	Fruit shape easily affects results
		Little influence by external factors	
	Impact	Simplicity of operator	Reflects the local firmness
		Relatively cheap	
	Acoustic	Reflects the overall firmness Suitable for samples with irregular shape or uneven size	Reduces detection accuracy by ambient noise or vibration during signal acquisition
	Ultrasonic	High sensitivity to the change in tissue density, lacuna, and water content in fruit	Needs calibration for varying acoustic characteristics of different fruit
Electromagnetic technology	Vis/NIR spectroscopy	Good detection effect on chemical components and physical properties	Point detection Lack of comprehensive spatial information
		No sample pretreatment	
	Optical properties	Reflects the scattering and absorption characteristics in fruit	Generates errors due to small values of the optical absorption and scattering coefficients
Electromagnetic technology	Spatially resolved spectroscopy	Detects the internal and external quality characteristics simultaneously	Different kinds of samples require different mathematical models
	Hyperspectral imaging	Provides continuous spectral information to detect a variety of components;	Expensive equipment
		Captures spectral and spatial information to analyze the internal and surface quality simultaneously;	Large amount of data and complicated data analysis
		Without sample pretreatment	Slow acquisition speed for data
	Raman spectroscopic	High sensitivity to chemical composition and can detect the molecular characteristics of trace substances;	Slow detection speed High cost of the equipment Requires high power laser and high sensitivity detector because of weak Raman scattering signals
		Enables distinguishing similar chemical compositions	
	X-ray	Provides high-resolution images for detailed quality analysis	Requires expensive instrument
		Visualizes internal defects and structural anomalies	Necessitates strict safety measures to protect operators from radiation exposure
			less suitable for assessing chemical compositions
	Time-domain NMR	Provides accurate data on the content and distribution of components such as water and protein	Slow detection speed
		Qualitative analysis of various inorganic substances and organic compounds	The cost of the equipment is expensive
Electrochemical sensor technology	Electronic nose and tongue	High sensitivity	Low accuracy by the external environment influence
		Relatively cheap	Only suitable for liquid samples using the electronic tongue
		Fast detection speed	High sensor requirements

**Table 3:** Recent applications of deep learning combined with nondestructive detection for tomato quality assessment.

Technique	Index	DL Model	RMSEP	Results	Reference
Vision-based tactile sensing	Firmness	CNN–LSTM		Acc = 0.846	[168]
	Ripeness		1.839	R^2^ = 0.795	
Machine vision	Ripeness	NVW-YOLOv8s		Acc = 0.914	[169]
	Quality grades	MobileNetV3		Acc = 0.9669	[170]
	Ripeness	MTD-YOLOv7		Acc = 0.866	[171]
	Phenotype	R-CNN		Acc = 0.95	[172]
	Maturity	DNN		Acc = 0.93	[157]
	Appearance grade	YOLOv4		Acc = 0.999	[173]
	Maturity	R-CNN		Acc = 0.921	[174]
	Maturity	DenseNet		Acc = 0.9126	[159]
	Quality grades	DSSAEs		Acc = 0.955	[39]
Vis/NIR spectroscopy	Ripeness	FCNN (350–1100 nm)		Acc = 0.993	[175]
	Geographical Origin	SAE-SSA-SVM (1000–2500 nm)		Acc = 0.956	[176]
Hyperspectral imaging	SSC	CNN-Transformer (900–1700 nm)	0.56	R^2^ = 0.84	[177]
	pH		0.12	R^2^ = 0.6	
	Firmness		0.94	R^2^ = 0.92	[100]
	SSC	RNN	0.19	R^2^ = 0.88	
	Lycopene	(980–1660 nm)	0.73	R^2^ = 0.94	
	TA		0.03	R^2^ = 0.87	
	SSC	Con1dResNet	0.018	R^2^ = 0.901	[164]
	Firmness	(400–1000 nm)		R^2^ = 0.532	
Raman spectroscopy	Quality	CNN		R^2^ = 0.946	[165]

Acc, Accuracy. TA, Titratable Acidity. FCNN, Multimodal fusion fully connected network. SAE-SSA-SVM, Stacked autoencoder-sparrow search algorithm-SVM. R-CNN, Region-Convolutional Neural Network. Con1dResNet, one-dimensional convolutional ResNet. DNN, Deep neural network. DenseNet, Densely connected convolutional networks. DSSAEs, Deep stacked sparse auto-encoders.

## Data Availability

No new data were created or analyzed in this study. Data sharing is not applicable to this article.
